# TCRγδ^+^CD4^−^CD8^−^ T Cells Suppress the CD8^+^ T-Cell Response to Hepatitis B Virus Peptides, and Are Associated with Viral Control in Chronic Hepatitis B

**DOI:** 10.1371/journal.pone.0088475

**Published:** 2014-02-14

**Authors:** Qintao Lai, Shiwu Ma, Jun Ge, Zuxiong Huang, Xuan Huang, Xiaotao Jiang, Yongyin Li, Mingxia Zhang, Xiaoyong Zhang, Jian Sun, William G. H. Abbott, Jinlin Hou

**Affiliations:** 1 Hepatology Unit and Key Lab for Organ Failure Research, Department of Infectious Diseases, Nanfang Hospital, Southern Medical University, No. 1838, North Guangzhou Avenue, Guangzhou, China; 2 Department of Infectious Diseases, Kunming General Hospital of PLA, Kunming, China; 3 Department of immunology, Basic Medicine School, Southern Medical University, Auckland City Hospital, Auckland, New Zealand; 4 The New Zealand Liver Transplant Unit, Auckland City Hospital, Auckland, New Zealand; University of Athens, Medical School, Greece

## Abstract

The immune mechanisms underlying failure to achieve hepatitis B e antigen (HBeAg) seroconversion associated with viral control in chronic hepatitis B (CHB) remain unclear. Here we investigated the role of CD4^−^CD8^−^ T (double-negative T; DNT) cells including TCRαβ^+^ DNT (αβ DNT) and TCRγδ^+^ DNT (γδ DNT) cells. Frequencies of circulating DNT cell subsets were measured by flow cytometry in a retrospective cohort of 51 telbivudine-treated HBeAg-positive CHB patients, 25 immune tolerant carriers (IT), 33 inactive carriers (IC), and 37 healthy controls (HC). We found that γδ DNT cell frequencies did not significantly change during treatment, being lower at baseline (*P* = 0.019) in patients with HBeAg seroconversion after 52 weeks of antiviral therapy (n = 20) than in those without (n = 31), and higher in the total CHB and IT than IC and HC groups (*P*<0.001). αβ DNT cell frequencies were similar for all groups. In vitro, γδ DNT cells suppressed HBV core peptide-stimulated interferon-γ and tumor necrosis factor-α production in TCRαβ^+^CD8^+^ T cells, which may require cell–cell contact, and could be partially reversed by anti-NKG2A. These findings suggest that γδ DNT cells limit CD8^+^ T cell response to HBV, and may impede HBeAg seroconversion in CHB.

## Introduction

Worldwide, more than 350 million people have chronic hepatitis B virus (HBV) infection. The serious sequelae of this disease include liver cirrhosis, liver failure, and hepatocellular carcinoma [1]. Hepatitis B e antigen (HBeAg) seroconversion, defined as the loss of serum HBeAg with development of anti-HBe, is a stage in the evolution of chronic HBV infection, which can occur either spontaneously or as a result of antiviral therapy. For HBeAg-positive patients with chronic hepatitis B (CHB), HBeAg seroconversion is one of the primary goals of antiviral treatment, as it is associated with control of viral load, cessation of liver inflammation, decreased risk for serious sequelae, and increased survival [2]. Unfortunately, most patients do not achieve HBeAg seroconversion on treatment, for reasons that are poorly understood.

Evidence that HBeAg seroconversion is the result of a host immune response to HBV has recently been summarized [3]. Genetic polymorphisms in IL-10 and IL-12 loci [4], and differences in serum IL-12 [5] and serum IL-21 [6] concentrations may predict HBeAg seroconversion. However, there is little information on the cellular immune mechanisms that might be responsible for these associations. These mechanisms need to be identified to allow optimizing treatment strategies in individual patients and developing novel immunotherapies to increase seroconversion rates. We focused on the CD4^−^CD8^−^ subset of regulatory T cells, also known as double-negative T (DNT) cells.

DNT cells are classified into αβ DNT and γδ DNT subsets, depending on whether their TCR is comprised of αβ or γδ chains. αβ DNT cells can regulate antigen-specific T-cell responses in both mice [7] and humans [8], [9], and HBeAg-activated αβDNT cells can inhibit the murine HBeAg-specific effector T-cell response [10]. γδ T cells, which are usually CD4- and CD8-negative, have a role in immunoregulation, including maintenance of oral tolerance [11], ocular tolerance [12], and hyporesponsiveness to tumors [13] in mice. Human γδ T cells also possess immune-suppressive capabilities [14], [15], [16], [17], [18]. Thus, DNT cells may involve in suppression of any cellular immune response that promotes HBeAg seroconversion. To investigate this possibility, we conducted a longitudinal study comparing DNT cell frequencies in patients with CHB who either did or did not undergo HBeAg seroconversion with antiviral therapy, and a cross-sectional comparison of DNT cell frequencies in subjects who were either HBeAg-positive or HBeAg-negative.

## Materials and Methods

### Human Subjects

This study was conducted according to the ethical guidelines of the 1975 Declaration of Helsinki, and was approved by the ethics committee of Nanfang Hospital. All subjects provided written informed consent.

HBeAg-positive patients with CHB for the longitudinal study (n = 51) were participating in either a phase IV, multi-center, open-label clinical trial of telbivudine (LdT 600 mg/d, n = 21, trial number CLDT600ACN07T ) or the Efficacy Optimization Research of Telbivudine Therapy trial (EFFORT, n = 30, trial number NCT00962533). From each patient, 20 mL of heparinized blood was taken at baseline and at 12, 24, and 52 weeks of LdT treatment. Patients were classified as responders (n = 20) if they achieved HBeAg seroconversion by treatment week 52, and as non-responders (n = 31) if they did not. Baseline characteristics of the patients in these two groups are compared in Table 1. Sixteen responders who received LdT consolidation therapy and maintained a durable response (HBeAg-negative, anti-HBe-positive, HBV DNA <300 copies/mL, and normal alanine aminotransferase (ALT)) for 48 weeks were followed up for another 52 weeks off treatment. Virologic relapse was defined as an increase of DNA level to >10,000 copies/mL [19]. The subjects for cross-sectional study included IT (n = 25), CHB (n = 51 from the above CHB), IC (n = 33) and HC (n = 37), who were recruited according to definition in AASLD criteria (see Methods S1) [2]. Their characteristics are summarized in Table 2.

**Table 1:** Clinical characteristics at baseline of subjects in the cohort receiving telbivudine therapy.

Groups	Responders	Non-responders	*P* values
Cases	20	31	
Gender, M/F	14/6	26/5	0.408b)
Age, yearsa)	28 (25–35)	27 (24–29)	0.250c)
ALT, IU/La)	166 (98–252)	116 (82–244)	0.370c)
AST, IU/La)	104 (53–129)	88 (57–146)	0.946c)
HBV DNA, log10 copies/mLa)	8.16 (7.32–8.72)	8.73 (8.59–9.03)	0.002c)
Genotype B/C	16/4	16/15	0.080b)

ALT, alanine aminotransferase; AST, Aspartate aminotransferase; HBV, hepatitis B virus; M/F: Male/female.

a) Data are median (interquartile range).

b) Compared by χ^2^ test (continuity correction).

c) Compared by Mann-Whitney *U*-test.

**Table 2:** Clinical characteristics of subjects in cross-sectional study.

Groups	IT	CHB	IC	HC
Cases	25	51	33	37
Gender, M/F	11/14	40/11	10/23	15/22
Age, yearsa)	26 (23–30)	27 (24–33)	29 (24–38)	24 (23–26)
ALT, IU/La)	27 (17–32)	127 (88–244)	19 (16.5–25)	13(8–22)
AST, IU/La)	25 (22–27)	99 (57–137)	24 (21–28)	20 (16.5–24)
HBV DNA, log10 copies/mLa)	7.80 (7.49–8.30)	8.62 (8.00–8.90)	<3.00(<3.00)	NA
HBeAg/anti-HBe	25/0	51/0	0/33	0/0

ALT, alanine aminotransferase; AST, Aspartate aminotransferase; HBeAg, HBV e antigen; HC, healthy controls; IC, inactive carriers; IT, immune tolerant carriers; M/F: Male/female; NA, not applicable.

a) Data are median (interquartile range).

### Synthetic Peptides

A panel of 26 18-mer peptides overlapping by 8 or 10 residues and covering the full HBV core open reading frame (Table S1 and S2) and HBV core18–27 (FLPSDFFPSV) were obtained (Sigma-Aldrich; St. Louis, MO, USA). 18-mer Peptides were dissolved in dimethyl sulfoxide (DMSO), pooled, and named the core peptides pool. An 18-mer HIV gag peptide (VDRFYKTLRAEQASQEVK) dissolved in DMSO was used as the negative control, while medium only as the blank control.

### Cell Isolation and Flow-cytometry Analysis

Isolation of peripheral blood mononuclear cells (PBMC) [6] and liver-infiltrating lymphocytes (LIL) [20], [21] was performed (see Methods S2). For analysis of the DNT cell subset frequency, cells were stained with anti-CD3-APC (UCTH1), anti-TCRγδ-FITC (11F2), anti-CD4-PE, anti-CD8-PE (all BD Biosciences, Pharmingen, San Jose, CA, USA) and anti-TCRαβ-PE-Cy7 (IP26; BioLegend, San Diego, CA, USA). Flow-cytometry analysis was performed using a FACS CanTo II flow cytometer and BD FACSDiva software (BD Biosciences).

Intracellular cytokine staining (ICS) was performed as previously described [6], [22], [23]. Briefly, 4×10^5^ PBMC were incubated with either the core peptide pool (each peptide at a final concentration of 1 µg/mL) plus anti-CD49d (1 µg/mL) and anti-CD28 (1 µg/mL), or with anti-CD3 (OKT, 5 µg/mL) plus anti-CD28 (1 µg/mL, BD Biosciences), or with phorbol-12-myristate-13-acetate (PMA, 50 ng/mL) plus calcium ionomycin (1 nmol/mL; Sigma-Aldrich) for 1 hour in 200 µL R10 (RPMI 1640 containing 2 nmol/mL glutamine, 10% FBS, 100 U/mL penicillin, and 100 µg/mL streptomycin) in a round-bottomed 96-well plate, followed by 1 µg/mL brefeldin A (BD Biosciences) for an additional 5 hours. The cells were harvested, stained with cell surface markers, fixed, permeabilized, and stained with either anti-interferon (IFN)-γ-PerCP Cy5.5, anti-tumor necrosis factor (TNF)-α-PE or isotype controls (BD Biosciences) for flow-cytometry analysis.

The frequency of a positive CD8^+^ T-cell response to core peptides in patients with CHB is usually <30% [23], [24], [25]. In this study, positive response was defined as greater production of either IFN-γ or TNF-α by core peptide-stimulated CD8^+^ T cells compared with either culture medium-stimulated or HIV gag peptide-stimulated CD8^+^ T cells. Patients with a positive CD8^+^ T-cell response were chosen for the T-cell suppression assays, transwell assays, and blocking assays.

### T-cell Suppression Assays

Because the proportion of CD4^+^ γδ T cells in the γδ T-cell pool is extremely low (<1%), the isolation of γδ DNT was carried out using magnetic beads (Miltenyi Biotech, Bergisch Gladbach, Germany) to remove CD8^+^ cells from PBMC, followed by positive selection of γδ T cells. γδ T(−) PBMC were obtained by direct removal of γδ T cells. The γδ DNT : γδ T(−) PBMC co-cultures were set up at a ratio of 1∶2 with a total of 4.5×10^5^ cells/well. After overnight incubation, both the γδ DNT : γδ T(−) PBMC and γδ T(−) PBMC were cultured with the core peptide pool, and the production of IFN-γ in TCRαβ^+^CD8^+^ T (CD8^+^ αβ T) cells was detected by ICS. Negative or blank controls were 4.5×10^5^ γδ T(−) PBMC cultured with or without the HIV gag peptide, respectively.

### Transwell Assays

Transwell assays were performed using cell-culture inserts with a 1 µm polycarbonate membrane to avoid direct cell contact between the top and bottom chambers in 96-well plates (Transwell; Corning Inc., Corning, NY, USA). The γδ T(−) PBMC (4.5×10^5^) were added to the bottom chamber, while γδ DNT cells (1.5×10^5^) were added to either the top or the bottom chamber. Production of IFN-γ and TNF-α in core peptide-stimulated CD8^+^αβ T cells in the bottom chamber was detected by ICS.

### Blocking Assays

For more sensitive detection, blocking assays were performed as previously described [24], [26], [27], with minor modifications. Briefly, 1×10^5^ γδ DNT cells were respectively incubated with anti-NKG2A (Z199, 10 µg/mL; Beckman Coulter Inc., Brea, CA, USA), anti-HLA-E(3D12, 10 µg/mL), or mouse IgG1 isotype (10 µg/mL) (both Biolegend) for 45 min at 37°C, and then co-cultured with 3×10^5^ γδ T(−)PBMC in the presence of the core peptides pool, anti-CD49d, and anti-CD28 (1 µg/mL). The gag peptide was the negative control. IL-2 (25 U/mL) was added on day 4. After 10 days, cells were resuspended, and stimulated again with peptides for 1 h, then Brefeldin A was added for a further 5 h. Production of IFN-γ and TNF-α in CD8^+^αβT cells was measured by flow-cytometry analysis.

PBMC from some patients were stained with anti-HLA-A2-PE (BD). The HLA-A2 positive patients were chose for the detection using PE-labeled pentamer complexed with HBV core 18–27 (Proimmune, Oxford, UK). With the method of blocking assays, cells were expanded and re-stimulated with HBV core18–27 (5 µg/mL), and stained with anti-CD3-FITC, anti-CD8-APC-Cy7, pentamer-PE, anti-IFN-γ-PerCP-Cy5.5 and anti-TNF-α-PE-Cy7.

### Statistical Analysis

Serum HBV DNA levels were normalized by log transformation. Box and whisker plots were used to show the median, interquartile range (IQR), and range. For independent group comparisons, we used the Kruskal-Wallis *H*-test to compare all groups and the Mann-Whitney *U*-test to compare groups by pairs. For related group comparisons, the Friedman test was used to compare all groups and the Wilcoxon test to compare groups by pairs. Spearman rank order correlation was used for correlation analyses. The χ^2^ test, logistic regression, and receiver-operating characteristic curves (ROC) analysis were used as appropriate, using either SPSS (version 13.0; SPSS Inc., Chicago, IL, USA), GraphPad Prism (version 5; GraphPad, San Diego, CA, USA) or SAS (SAS Inc., Cary, NC, USA). For ROC analysis, an area under the curve (AUC) of 1.0 is characteristic of an ideal test, whereas AUC <0.5 indicates a test of no diagnostic value. A (two-tailed) *P*-value of <0.05 was deemed statistically significant.

## Results

### Association between Frequencies of γδ DNT Cells and HBeAg Seroconversion in Antiviral Therapy

**Figure 1:**
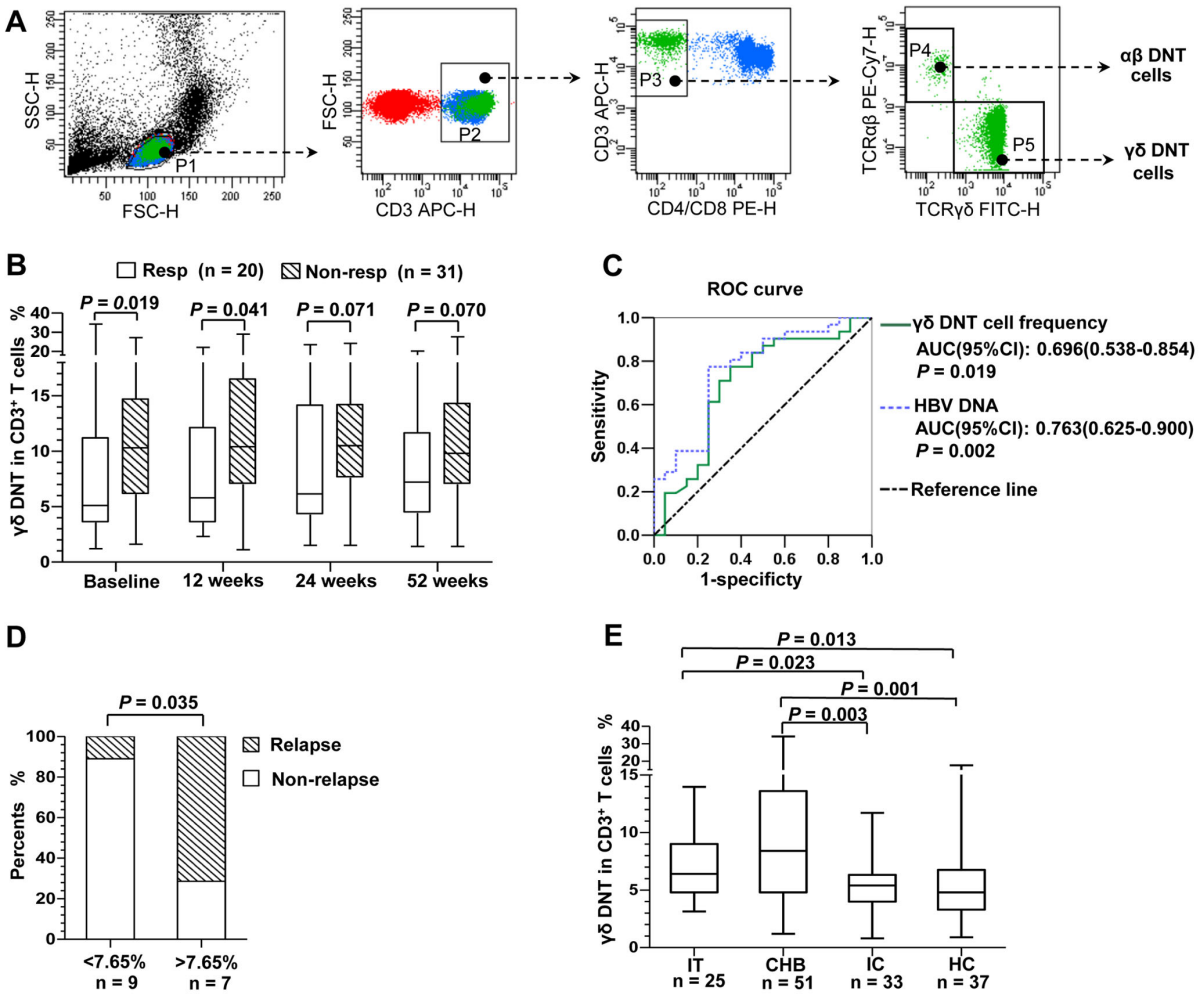
Comparison of the frequencies of DNT cell subsets in longitudinal and cross-sectional studies. (A) Flow-cytometry dot plots show the strategy for identifying the αβ DNT cells and γδ DNT cells. (B) The frequencies of γδ DNT cells in peripheral blood CD3^+^ T cells were compared for responders (Resp, n = 20) and non-responders (Non-resp, n = 31) at different time points during 52 weeks of telbivudine therapy using the Mann-Whitney U test by pairs. (C) Receiver-operating characteristic curve analysis shows that either baseline γδ DNT cell frequencies or HBV DNA levels predicts the non-responders after 52 weeks of telbivudine therapy. (D) The virologic relapse rates after 52 weeks off treatment were compared between the patients with baseline γδ DNT cell frequency of >7.65% (n = 7) and <7.65% (n = 9) using χ^2^ test (Fisher’s exact test). (E) The frequencies of γδ DNT cells were compared in the IT, CHB, IC and HC groups using the Mann-Whitney *U* compared groups by pairs. (B, D) Data plots show the median, interquartile range (IQR), and range. αβ DNT cells, TCRαβ^+^CD4^−^ CD8^−^ T cells; γδ DNT cells, TCRγδ^+^CD4^−^CD8^−^ T cells; AUC, area under the curve; CI, confidence interval; CHB, chronic hepatitis B; DNT cells, double-negative T cells; HC, healthy controls; IC, inactive carriers; IT, immune tolerant carriers.

In longitudinal study, HBeAg-postive patients treated with telbivudine (LdT) were classified as responders (n = 20) if they achieved HBeAg seroconversion by treatment week 52, and as non-responders (n = 31) if they did not. Circulating frequencies of αβ DNT cells and γδ DNT cells were examined (Figure 1A) at baseline, 12, 24, and 52 weeks. There were no significant changes in frequencies of the two DNT cell subsets during 52 weeks of treatment, and no significant differences in frequencies of αβ DNT cells between the two groups (Figure 1B; Figure S1A). Frequencies of γδ DNT cells were higher in non-responders than in responders at baseline (median [IQR]: 10.3% [6.1–14.7%] vs 5.1% [3.6–11.2%], respectively; *P* = 0.019) and at 12 weeks (10.5% [7.0–16.5%] vs 5.8% [3.7–12.1%]; *P* = 0.041).

Univariate logistic regression showed that HBeAg seroconversion was associated with low baseline frequencies of both γδ DNT cells (*P* = 0.04) and HBV DNA (*P* = 0.005). Multivariate analysis identified a significant effect of HBV DNA (*P* = 0.008), and a trend towards an independent effect of γδ DNT cell frequency (*P* = 0.08). No significant interaction was found between these variables (*P* = 0.77). There were no significant correlations between frequencies of γδ DNT cells and either the serum HBV DNA levels, ALT, or aspartate aminotransferase (AST), or age of the CHB patients at baseline (Table S3).

In this cohort, ROC analysis showed that either baseline γδ DNT cell frequency or baseline serum HBV DNA level predicted the non-responders after 52 weeks of LdT therapy (Figure 1C). The optimal cut-off value for prediction was baseline γδ DNT cell frequency of 7.65% of CD3-positive PBMC, which had a sensitivity of 70.0% and specificity of 71.0%. In light of that quite a few patients who achieve HBeAg seroconversion induced by nucleotide analogues have virologic relapse off treatment [19], we observed the association between baseline γδ DNT cell frequency and viral control after 52 weeks off therapy. Sixteen patients received an average of 112 Weeks (rang 104–163 weeks) of LdT therapy before off-therapy. In the cohort of patients with baseline γδ DNT cell frequency >7.65%, five of seven patients had a virologic relapse after 52 weeks off treatment, whereas only one of nine patients with baseline γδ DNT cell frequency <7.65% had a virologic relapse (*P* = 0.035, Figure 1D). All patients (n = 4) who had recurrence of HBeAg had a high frequency of γδ DNT cells, with rates for the four patients being 10.4%, 11.3%, 14.2%, and 17.8%, respectively.

Collectively, these data indicate that a high frequency of γδ DNT cells is associated with failure to achieve durable HBeAg seroconversion induced by LdT therapy.

### Association between Frequencies of γδ DNT Cells and Serum HBeAg Status in Cross-sectional Comparison

To investigate whether frequency of γδ DNT cells is associated with spontaneous HBeAg seroconversion, we compared CHB with inactive carriers (IC), immune tolerant carriers (IT), and healthy controls (HC) at baseline. Frequencies of γδ DNT cells in the two HBeAg-positive groups (IT 6.4% [4.8–9.0%] and CHB 8.4% [4.8–13.6%]) were significantly higher than in either the IC (5.4% [4.0–6.3%]) or HC (4.8% [3.3–6.8%]) groups (Figure 1E). There were no significant differences in γδ DNT cell frequency between the CHB and IT groups, or between the IC and HC groups. These data indicate that a low γδ DNT cell frequency is associated with a state that is more likely to lead to spontaneous HBeAg seroconversion.

### Suppression of Anti-HBV CD8^+^ T cell Response by γδ DNT Cells

Together, the results of the clinical studies imply that a high frequency of γδ DNT cells is not good for viral control in CHB. Thus, we investigated whether γδ DNT cells suppress the HBV-specific CD8^+^ T-cell responses that are known to inhibit HBV replication [28].

**Figure 2:**
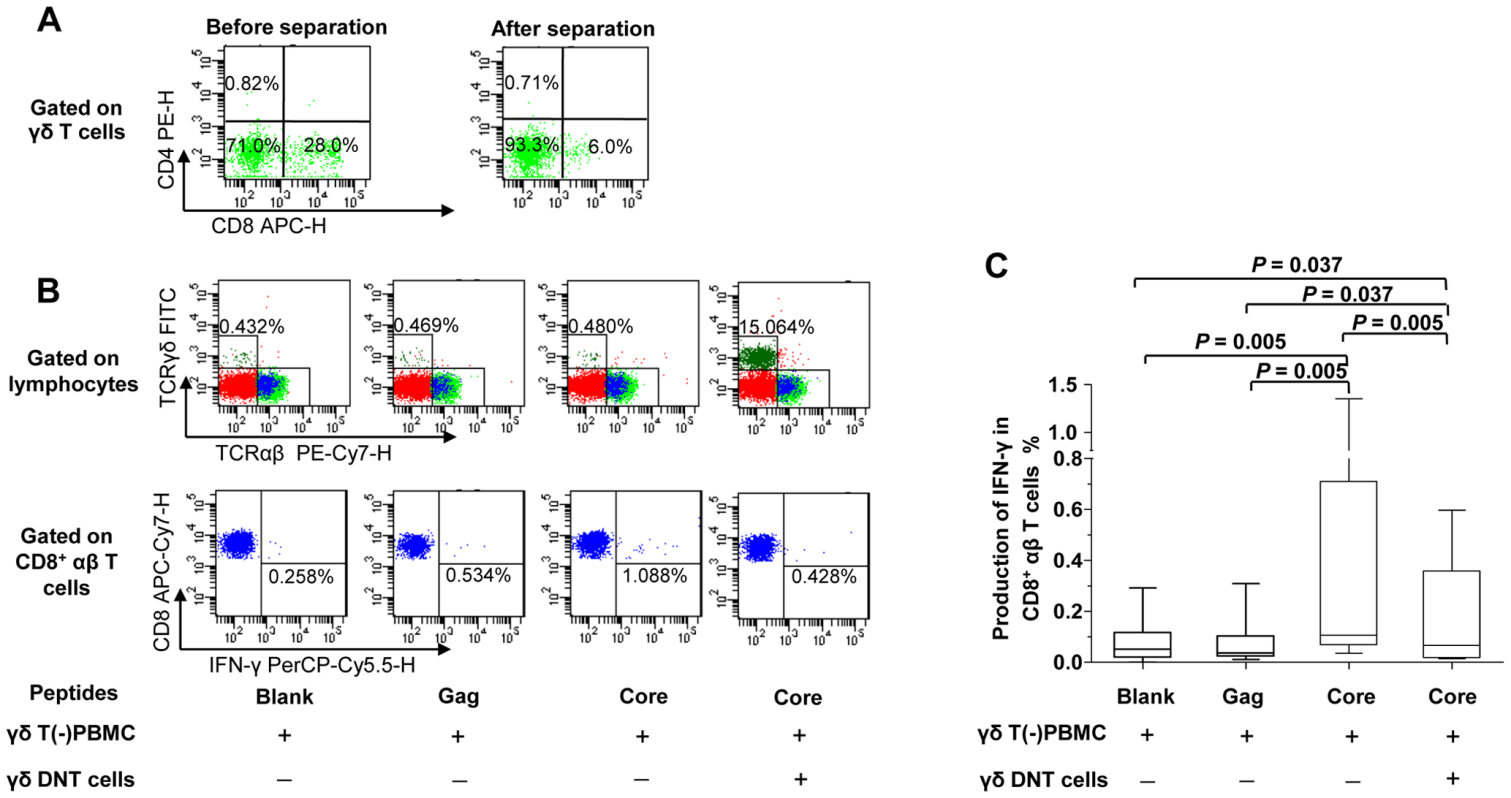
γδ DNT cells suppress HBV antigen-specific CD8^+^ αβ T-cell responses. (A) One representative example shows the purity of γδ DNT cells in a γδ T-cell pool before and after separation. (B) One representative result shows that addition of γδ DNT cells reduced HBV core peptide-induced production of IFN-γ in CD8^+^ αβ T cells. A gag peptide was used as the negative control, while medium only as the blank control. (C) The pooled results from ten patients with CHB were compared using Wilcoxon test by pairs. (blank, medium only; core, HBV core peptides pool; gag, HIV gag peptide). γδ T(−)PBMC, γδ T cell-deleted-PBMC; γδ DNT cells, CD8-deleted-γδT cells; CD8^+^ αβ T cells, TCRαβ^+^CD8^+^ T cells; CHB, chronic hepatitis B; DNT cells, double-negative T cells; IFN, interferon; PBMC, peripheral blood mononuclear cells.

In the T-cell suppression assay, the median purity of isolated γδ DNT cells within the γδ T cell compartment (Figure 2A) was 89.7% (range 86.1–96.3%, n = 10). Addition of γδ DNT cells to the core peptide-stimulated cultures reduced the frequency of IFN-γ-secreting CD8^+^αβ T cells (*P* = 0.005) (Figure 2B and C). In addition, from four patients with CHB, co-cultures of γδ T cells with a γδ DNT/γδ T frequency of >80% and γδ T(−) PBMC at ratios of 0∶16, 1∶16, 1∶4, 1∶2, and 1∶1 showed that a decreasing frequency of non-antigen specific, IFN-γ-producing cells in the CD8^+^ lymphocytes cultures (*P* = 0.007), and decreasing proliferation of CD8^+^ lymphocytes were accompanied by an increasing proportion of γδ T cells, which was dose-dependent (*P* = 0.003) (Figure S2A–F). However, there were no similar observations from three healthy donors (Figure S2G and H). It indicated that the suppressive function of γδ T cells was associated with the condition of chronic HBV infection.

### Mechanisms Associated with the Suppression by γδ DNT Cells

**Figure 3:**
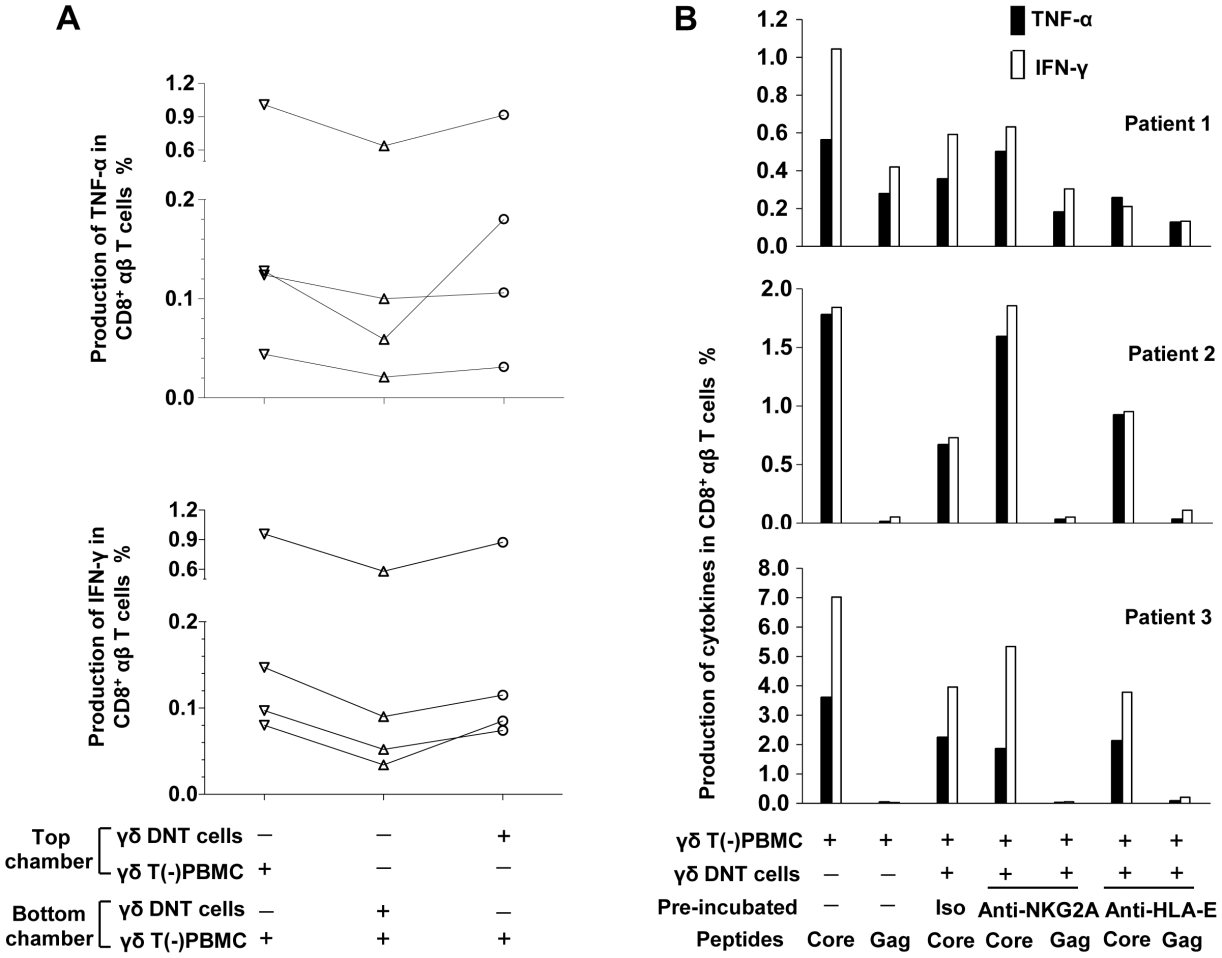
Mechanisms associated with the suppression by γδ DNT cells. (A) The suppression of HBV core peptide-induced production of TNF-α or IFN-γ in CD8^+^αβ T cells could be partly abrogated by inserting a membrane between the CD8^+^ αβ T cells and the γδ DNT cells in a transwell assay of cells from four patients with CHB. Lines link the data from the same patients. (B) γδ DNT cells were isolated and pre-incubated with either anti-NKG2A, anti-HLA-E, or mouse IgG1 isotype before being co-cultured with γδ T(−)PBMC. After 10 days of stimulation with peptides, production of TNF-α (black bars) and IFN-γ (blank bars) in CD8^+^αβ T cells was detected. Data from three patients are shown (core, HBV core peptides pool; gag, HIV gag peptide). DNT cells, double-negative T cells; IFN, interferon; PBMC, peripheral blood mononuclear cells; TNF, tumor necrosis factor.

**Figure 4:**
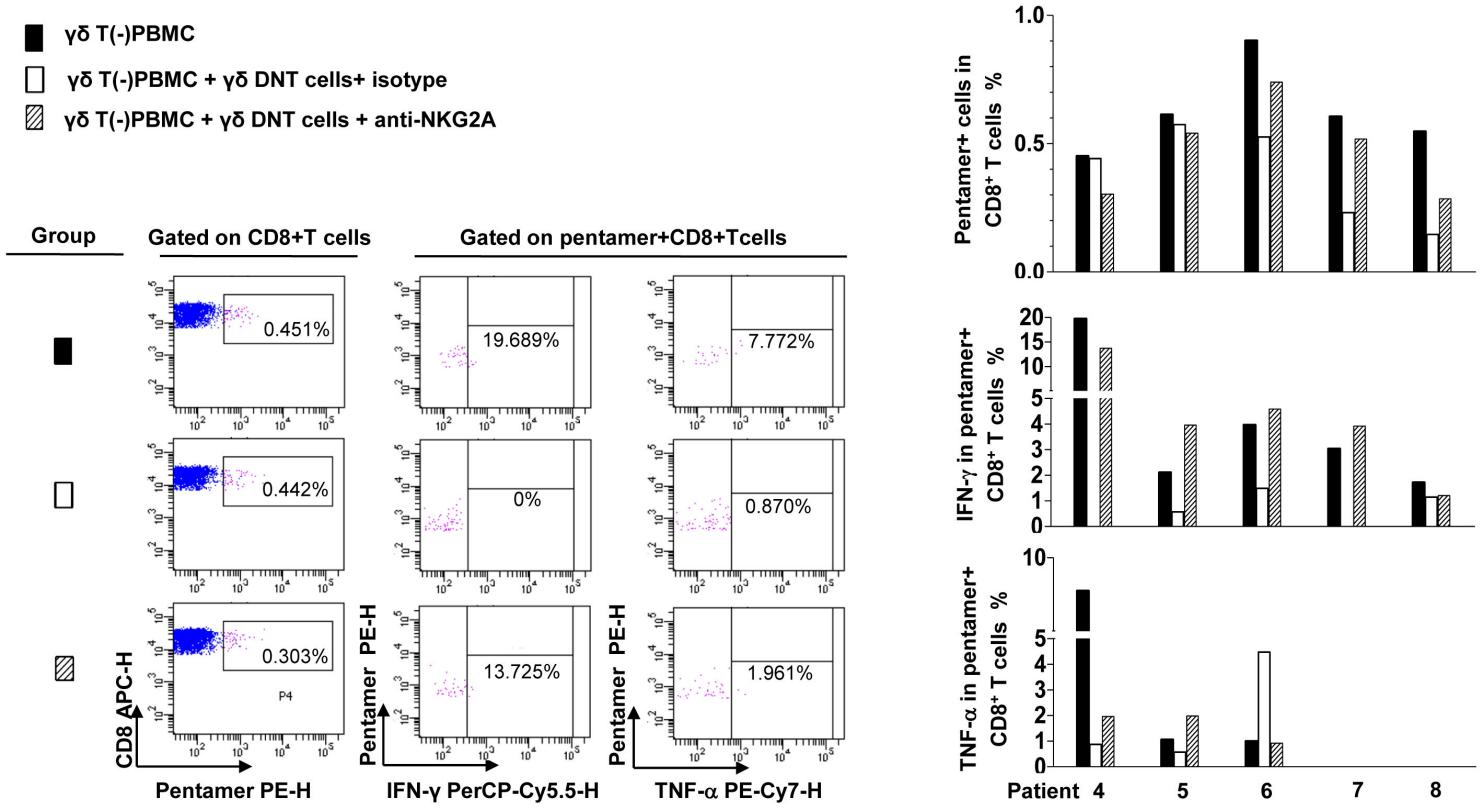
Influence of γδ DNT cells on HBV-specific pentamer-positive CD8^+^ T cells. γδ DNT cells were isolated from HLA-A2 positive patients with CHB and pre-incubated with either anti-NKG2A (diagonal stripes bars) or mouse IgG1 isotype (blank bars) before being co-cultured with γδ T(−)PBMC. The group with γδ T(−)PBMC only (black bars) was also compared. Dot plots from one representative patient show the strategy for identifying the frequency of HBV pentamer-positve CD8^+^ T cells, and the production of IFN-γ and TNF-α in pentamer-positive CD8^+^ T cells after 10 days of stimulation with HBV core18–27. Data from five patients are shown.

In the transwell assays, production of both TNF-α and IFN-γ by core peptide-stimulated γδT(−)PBMC increased when they were separated from γδ DNT cells by inserting a membrane (Figure 3A), indicating that suppression by γδ DNT cells may require cell–cell contact. In the CHB group, few γδ DNT cells expressed cell-surface CTLA-4, FasL, PD-L1, or LAG-3, and PMA-stimulated γδ DNT cells produced almost no TGF-β or IL-10 (data not shown), implying that these molecules may not directly mediate the suppression by γδ DNT cells. More than half of γδ DNT cells expressed NKG2A, and the frequency of NKG2A expression was higher on γδ DNT cells than on αβ DNT cells, CD4^+^ αβ T cells, NK cells, or CD8^+^ γδ T cells. There was no significant difference between CHB and HC in the frequency of γδ DNT cells expressing NKG2A (Figure S3). Interestingly, HLA-E, the ligand of NKG2A, was expressed by all of these T-cell subsets, and the frequency of HLA-E expression on γδ DNT cells was higher than on both αβ DNT cells and CD8^+^ αβ T cells. Moreover, frequency of HLA-E in CD8^+^ αβ T cells was higher in CHB and IT groups compared with HC (Figure S4). To investigate whether this NKG2A-HLA-E pathway is involved in the suppression by γδ DNT cells, we used an antibody to block NKG2A or HLA-E expressed on γδ DNT cells. Figure 3B (data derived from Figure S5) shows that suppression of both core peptide pool-induced TNF-α-secreting and IFN-γ-secreting CD8^+^ αβ T cells by γδ DNT cells was partly reversed in three patients after pre-incubation of γδ DNT cells with anti-NKG2A but not anti-HLA-E or IgG1 isotype. Furthermore, we investigated the influence of γδ DNT cells on the HBV-specific pentamer-positive CD8^+^ T cells. As shown in Figure 4, the frequency of HBV-specific pentamer-positive CD8^+^ T cells and the production of IFN-γ or TNF-α in these T cells decreased when γδ DNT cells were added. And such decreases can be partially reversed when γδ DNT cells were pre-incubated with anti-NKG2A in some patients.

### Association between Intrahepatic γδ DNT Cells and HBeAg Seroconversion

**Figure 5:**
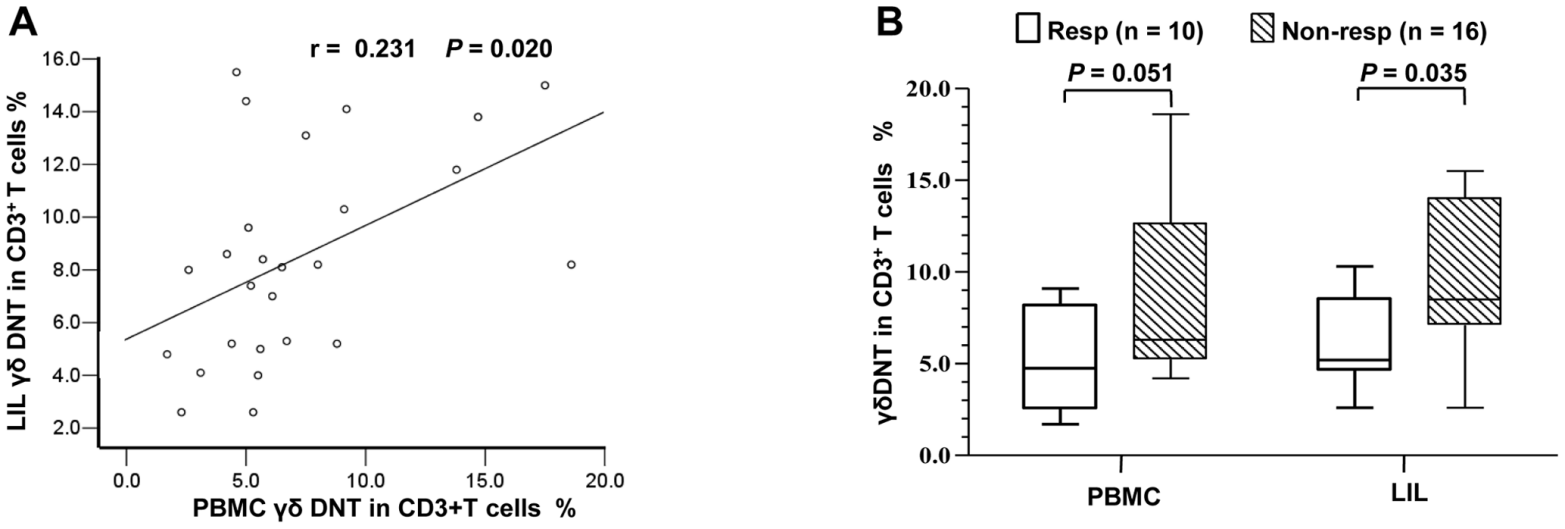
Frequencies of γδ DNT cell subsets in LIL and PBMC. LIL and PBMC were obtained from 26 patients with CHB at the same time after 104 weeks of telbivudine treatment. (A) Spearman’s correlation analysis between γδ DNT cell frequencies in LIL and PBMC. (B) Comparison of the frequencies of γδ DNT cells between LIL and PBMC using A Wilcoxon test, and between responders (Resp, n = 10) and non-responders (Non-resp, n = 16) using Mann-Whitney *U* test. CHB, chronic hepatitis B; DNT cells, double-negative T cells; LIL, liver-infiltrating lymphocytes; PBMC, peripheral blood mononuclear cells.

Because the HBV-specific CD8^+^ T-cell response mainly occurs in the liver, which is the site of HBV replication, we investigated whether the frequency of circulating γδ DNT cells reflects the frequency in the intrahepatic compartment. LIL and PBMC were obtained from 26 patients with CHB after 104 weeks of LdT treatment (10 responders and 16 non-responders). The characteristics of these patients at 104 weeks are compared between responders and non-responders in Table S4. There was no significant difference of Knodell necroinflammatory scores or Ishak fibrosis scores between these two groups. The γδ DNT cells were identified using a modified strategy (Figure S6). There was a significant correlation between frequencies of γδ DNT cells in LIL and PBMC (r = 0.231, *P* = 0.020, Figure 5A), both of which appeared similar (*P* = 0.107, Figure 5B). The frequency of γδ DNT cells was higher in LIL from non-responders relative to responders (8.5%, [7.1–14.0%] vs 5.2%, [4.6–8.5%]; *P* = 0.035). The higher frequency of γδ DNT cells in PBMC from non-responders relative to responders approached statistical significance (6.3%, [5.2–12.7%] vs 4.8% [2.5–8.2%]; *P* = 0.051).

## Discussion

In this study, we tested the hypothesis that DNT cells are involved in failure to achieve HBeAg seroconversion associated with viral control, which is supported by several clinical observations. Firstly, the baseline frequency of γδ DNT cells in PBMC from the patients who seroconverted after antiviral therapy was half that of patients who did not seroconvert. Secondly, when antiviral therapy was discontinued, virologic relapse and recurrence of HBeAg tended to occur in those patients who had a high baseline frequency of γδ DNT cells. Thirdly, the frequency of intrahepatic γδ DNT cells was higher in patients who didn’t achieve HBeAg seroconversion after 104 weeks of antiviral treatment. Finally, the frequency of γδ DNT cells was higher in the HBeAg-positive patients relative to IC, who are known to have a durable spontaneous HBeAg seroconversion. In the multivariate analysis of longitudinal data, the influence of baseline γδ DNT cell frequency on HBeAg seroconversion was not statistically independent of the influence of baseline HBV DNA level, but there was no correlation between γδ DNT cell frequency and HBV DNA level. It is difficult to determine whether these two parameters are reflections of the same underlying mechanism for HBeAg seroconversion.

Although no published evidence has shown that HBeAg seroconversion is caused by the activation of CD8^+^ T-cell responses to HBV antigens, there is a strong association between these two events [28], [29], [30]. Our in vitro data showed that γδ DNT cells have the ability to suppress anti-HBV-specific CD8^+^ T-cell responses. This raises the possibility that suppression of anti-viral CD8^+^ T-cell responses by a high level of γδ DNT cells contributes to failure to control HBV replication and achieve HBeAg seroconversion. This in vitro data also adds to the list of known mechanisms that contribute to CD8^+^ T cell hyporesponsiveness in patients with CHB, including impaired function of dendritic cells [31], increased numbers of CD4^+^CD25^+^ regulatory T cells [32], high antigen loads [33], high levels of expression of PD-1 by exhausted HBV-specific CD8^+^ T cells [26], and deletion of HBV-specific CD8^+^ T cells by NK cells [34].

γδ T cells are known to have a wide range of functions, including immune protection against pathogens in the early γδ T-cell response and immunoregulation in some late γδ T-cell responses [35], [36]. The impaired IFN-γ production and cytotoxicity of γδ T cells in persistent HBV infection have been reported previously [37]. Our study provides new insight into the immunoregulation of γδ T cells in CHB. The in vitro data indicates that suppression of HBV peptide-specific CD8^+^ T-cell responses by γδ DNT cells requires cell-cell contact, and is partially mediated by the NKG2A signaling pathway. These results are in contrast to a study of human γδ T cells expanded from breast tumors [15], but are consistent with a study of intraepithelial γδ T cells in patients with celiac disease [16]. As reviewed, NKG2A, an inhibitory receptor expressed by NK cells and CD8^+^ T cells, plays an important role in HIV, HCV [38], [39], and HBV [40] infections. NKG2A also mediates suppressive signaling in human γδ T cells [16], [41]. In the current study, we found that most γδ DNT cells expressed NKG2A, and the frequency of HLA-E in CD8^+^ αβ T cells was higher in patients with CHB. These findings suggest that two mechanisms may enhance γδ DNT cell-mediated suppression of anti-HBV CD8^+^ T-cell responses. The first is an increase in the number rather than the activity of γδ DNT cells. The second is an increase in the expression of HLA-E on CD8^+^ αβ T cells from HBeAg-positive subjects, which may be similar to the findings of up-regulation of HLA-E in HCV infections [42].

Pro-inflammatory cytokine or TCRγδ activation can augment γδ T-cell frequency in peripheral blood [35]. However, it seems unlikely that hepatic inflammation or viral factors are responsible for the increase in γδ DNT cell frequency seen in our study, as there were no significant changes in γδ DNT cell frequency with the decrease of ALT or serum viral components including HBV DNA, HBeAg, and hepatitis B surface antigen (HBsAg) during treatment, and there was no significant correlation between these variables at baseline. Thus, the mechanisms that regulate the circulating frequency of γδ DNT cells in CHB deserve further investigation.

We also analyzed frequencies of CD4^+^/CD8^+^ γδ T cells both in longitudinal and cross-sectional study. There was no significant difference in CD4^+^/CD8^+^ γδ T-cell frequencies between the different groups (Figure S1B and data not shown). CD4^+^ γδ T cells make up a small proportion of γδ T cells and most of CD4^+^/CD8^+^ γδ T cells were CD8^+^ γδ T cells. Little is known about the different properties between human CD8^+^ γδ T cells and γδ DNT cells [16]. A recent report showed that the absolute number of CD8^+^ γδ T cells did not change with age, whereas γδ DNT cells decreased in healthy aged people [43], Interestingly, in contrast, the cumulative lifetime incidence of HBeAg seroclearance increases with age [44]. Although we found higher frequencies of CD127, NKG2A, and HLA-DR, and lower frequencies of CD45 RA on γδ DNT cells compared with CD8^+^ γδ T cells (unpublished data), the different functions of these two subsets in CHB require further investigation.

In conclusion, a high frequency of γδ DNT cells is associated with failure to control HBV. A low baseline frequency of γδ DNT cells in PBMC predicts HBeAg seroconversion during LdT therapy for CHB. In vitro data showing that γδ DNT cells suppress CD8^+^ T cell responses to HBV antigens raise the possibility that the NKG2A-HLA-E signaling pathway may be a target for immunotherapy.

## Supporting Information

Figure S1 **Comparison of the frequencies of αβ DNT cells and CD4^+^/CD8^+^ γδ T cells in the longitudinal study.**
(TIF)

Figure S2 **Influence of different frequencies of γδ T cells on the non-specific CD8^+^ T-cell response.** CHB patients with a γδ DNT/γδ T-cell frequency of >80% were recruited into this study. γδ T cells were isolated from PBMC, and γδ T : γδ T(−)PBMC overnight co-cultures were set up at ratios of 0∶16, 1∶16, 1∶4,1∶2, and 1∶1 with a total of 4.5×10^5^ cells/well, and incubated in the presence of anti-CD3 and anti-CD28 antibodies to test the production of IFN-γ by ICS. (A,B) An individual experiment shows the influence of an increasing frequency of (A) γδ T cells (labeled “P1”, γδ DNT/γδ T = 82.9%) on (B) the production of IFN-γ (“P2”) in CD8^+^ lymphocytes stimulated with anti-CD3 and anti-CD28. In the proliferation test, γδ T(−)PBMC labeled with 1.5 µmol/L CFSE were co-cultured with γδ T cells in the presence of anti-CD3 and anti-CD28 antibodies for 3 days, and then stained with anti-CD8-PE for analysis of the proliferation of CD8^+^ lymphocytes by flow cytometry. (C,D) An individual experiment shows the influence of an increasing frequency of (C) γδ T cells (γδ DNT/γδ T cells = 90.3%) on (D) the proliferation of CD8^+^ lymphocytes stimulated with anti-CD3 and anti-CD28. “P3” indicates the unlabeled γδ T cells, “P4” the γδ T(−)PBMC labeled with CFSE, and “P5” the proliferating CD8^+^ lymphocytes. (E,F) Data from four patients with CHB were analyzed by the Friedman test. (G,H) Data from three healthy donors were also shown. CHB, chronic hepatitis B; DNT cells, double-negative T cells; ICS, intracellular cytokine staining.
(TIF)

Figure S3 **Expression of NKG2A on γδ DNT cells.** PBMC from CHB patients and HC were stained with anti-CD3-APC-Cy7, anti-TCR-γδ-FITC, anti-CD4-PE-Cy7, anti-CD8-PerCP, anti-CD56-APC and anti-NKG2A-PE. (A) Expression of NKG2A on γδ DNT, CD8^+^γδ T, αβ DNT, CD4^+^αβ T cells, and NK cells were measured and compared between (B) different lymphocyte subsets or (C) between the CHB and HC groups. CHB, chronic hepatitis B; DNT cells, double-negative T cells; HC, healthy controls.
(TIF)

Figure S4 **Expression of HLA-E on γδ DNT cells.** PBMC were stained with anti-CD3-APC-Cy7, anti-TCRγδ-FITC, anti-CD4-PE-Cy7, anti-CD8-PerCP, and anti-HLA-APC. Expression of HLA-E on γδ DNT, CD8^+^ γδ T cells, αβ DNT cells, and CD8^+^ αβ T cells was (A) measured in HC and CHB relative to the isotype control and (B) compared in the different T-cell subsets. (C,D) Expression of HLA-E on either (C) γδ DNT or (D) CD8^+^ αβ T cells was compared in the IT, CHB, and HC groups. CHB, chronic hepatitis B; DNT cells, double-negative T cells; HC, healthy controls; IT, immune tolerant carriers.
(TIF)

Figure S5 **γδ DNT cell-mediated suppression of cytokine production by core peptide-stimulated CD8^+^αβ T cells is partially mediated by NKG2A.** The plots were gated on CD8^+^αβ T cells. DNT cells, double-negative T cells.
(TIF)

Figure S6 **Strategy for gating the αβ DNT cells and γδ DNT cells from LIL.** DNT cells, double-negative T cells; LIL, liver-infiltrating lymphocytes.
(TIF)

Table S1 **The GenBank accession numbers of the sequences used to identify a panel of 26 18-mer peptides overlapping by 8 or 10 residues and covering the full HBV core open reading frame.**
(DOC)

Table S2 **The amino acid sequence of core peptides.**
(DOC)

Table S3 **Spearman’s correlation analyses showing associations between the frequencies of γδ DNT cells and the clinical characteristics of the CHB patients at baseline n = 51).**
(DOC)

Table S4 **Clinical characteristics of subjects in the cohort receiving telbivudine therapy at 104 Weeks.**
(DOC)

Methods S1 **Entry criteria for study subjects.**
(DOCX)

Method S2 **Isolation of peripheral blood mononuclear cells and liver-infiltrating lymphocytes.**
(DOCX)
