# Supplementary figures and images for: TCRγδ+CD4−CD8− T Cells Suppress the CD8+ T-Cell Response to Hepatitis B Virus Peptides, and Are Associated with Viral Control in Chronic Hepatitis B
Source: PLoS One. 2014 Feb 14;9(2):e88475. doi: 10.1371/journal.pone.0088475 (PMC3925121; doi:10.1371/journal.pone.0088475)

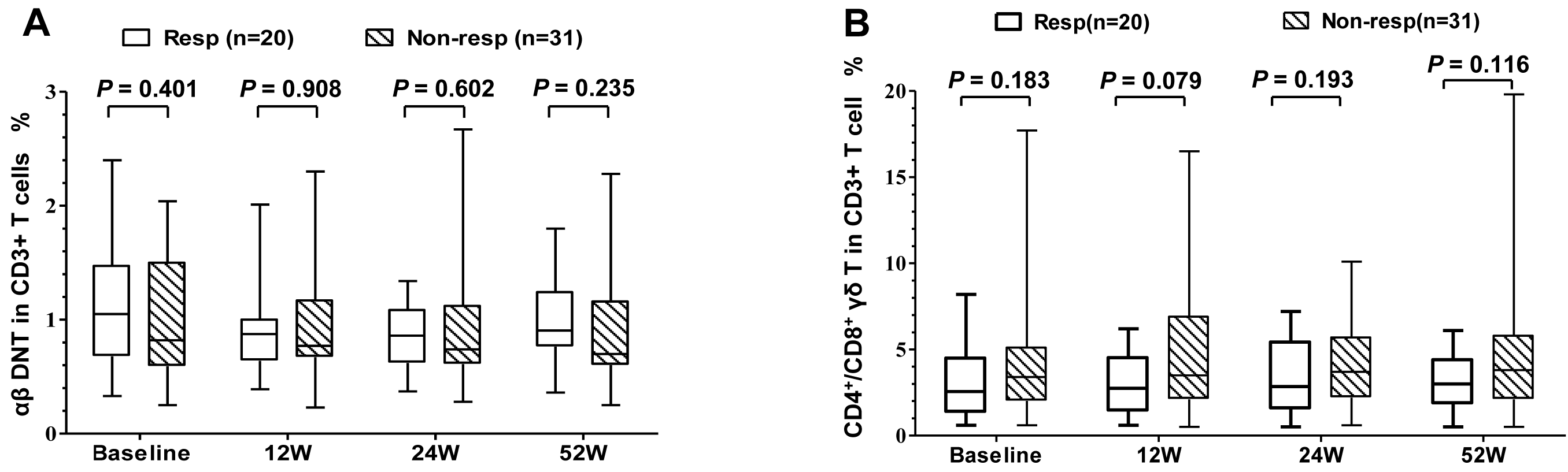

Supplement: Figure S1 — Comparison of the frequencies of αβ DNT cells and CD4+/CD8+ γδ T cells in the longitudinal study. (TIF) [file pone.0088475.s001.tif]

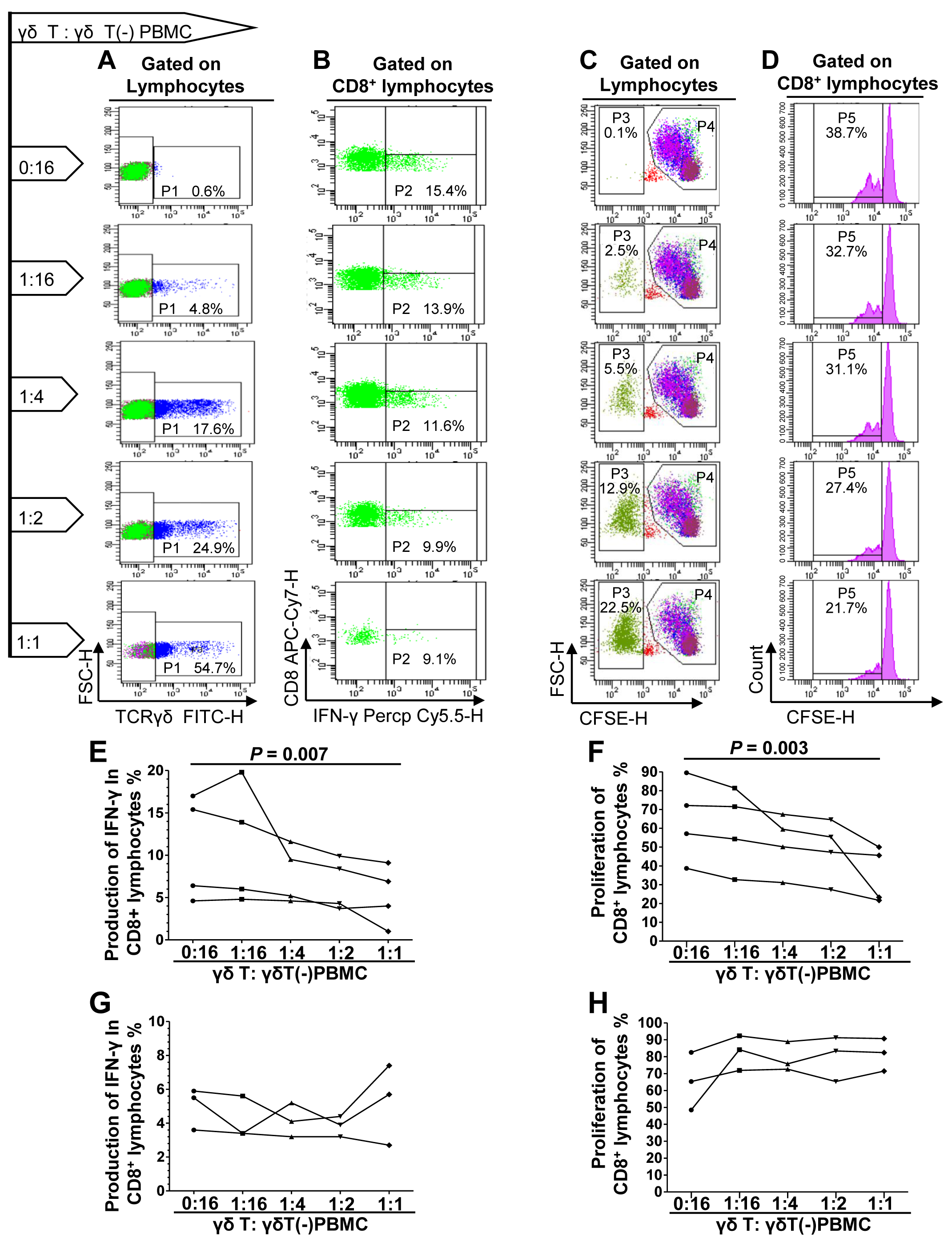

Supplement: Figure S2 — Influence of different frequencies of γδ T cells on the non-specific CD8+ T-cell response. CHB patients with a γδ DNT/γδ T-cell frequency of >80% were recruited into this study. γδ T cells were isolated from PBMC, and γδ T : γδ T(−)PBMC overnight co-cultures were set up at ratios of 0∶16, 1∶16, 1∶4,1∶2, and 1∶1 with a total of 4.5×10^5^ cells/well, and incubated in the presence of anti-CD3 and anti-CD28 antibodies to test the production of IFN-γ by ICS. (A,B) An individual experiment shows the influence of an increasing frequency of (A) γδ T cells (labeled “P1”, γδ DNT/γδ T = 82.9%) on (B) the production of IFN-γ (“P2”) in CD8+ lymphocytes stimulated with anti-CD3 and anti-CD28. In the proliferation test, γδ T(−)PBMC labeled with 1.5 µmol/L CFSE were co-cultured with γδ T cells in the presence of anti-CD3 and anti-CD28 antibodies for 3 days, and then stained with anti-CD8-PE for analysis of the proliferation of CD8+ lymphocytes by flow cytometry. (C,D) An individual experiment shows the influence of an increasing frequency of (C) γδ T cells (γδ DNT/γδ T cells = 90.3%) on (D) the proliferation of CD8+ lymphocytes stimulated with anti-CD3 and anti-CD28. “P3” indicates the unlabeled γδ T cells, “P4” the γδ T(−)PBMC labeled with CFSE, and “P5” the proliferating CD8+ lymphocytes. (E,F) Data from four patients with CHB were analyzed by the Friedman test. (G,H) Data from three healthy donors were also shown. CHB, chronic hepatitis B; DNT cells, double-negative T cells; ICS, intracellular cytokine staining. (TIF) [file pone.0088475.s002.tif]

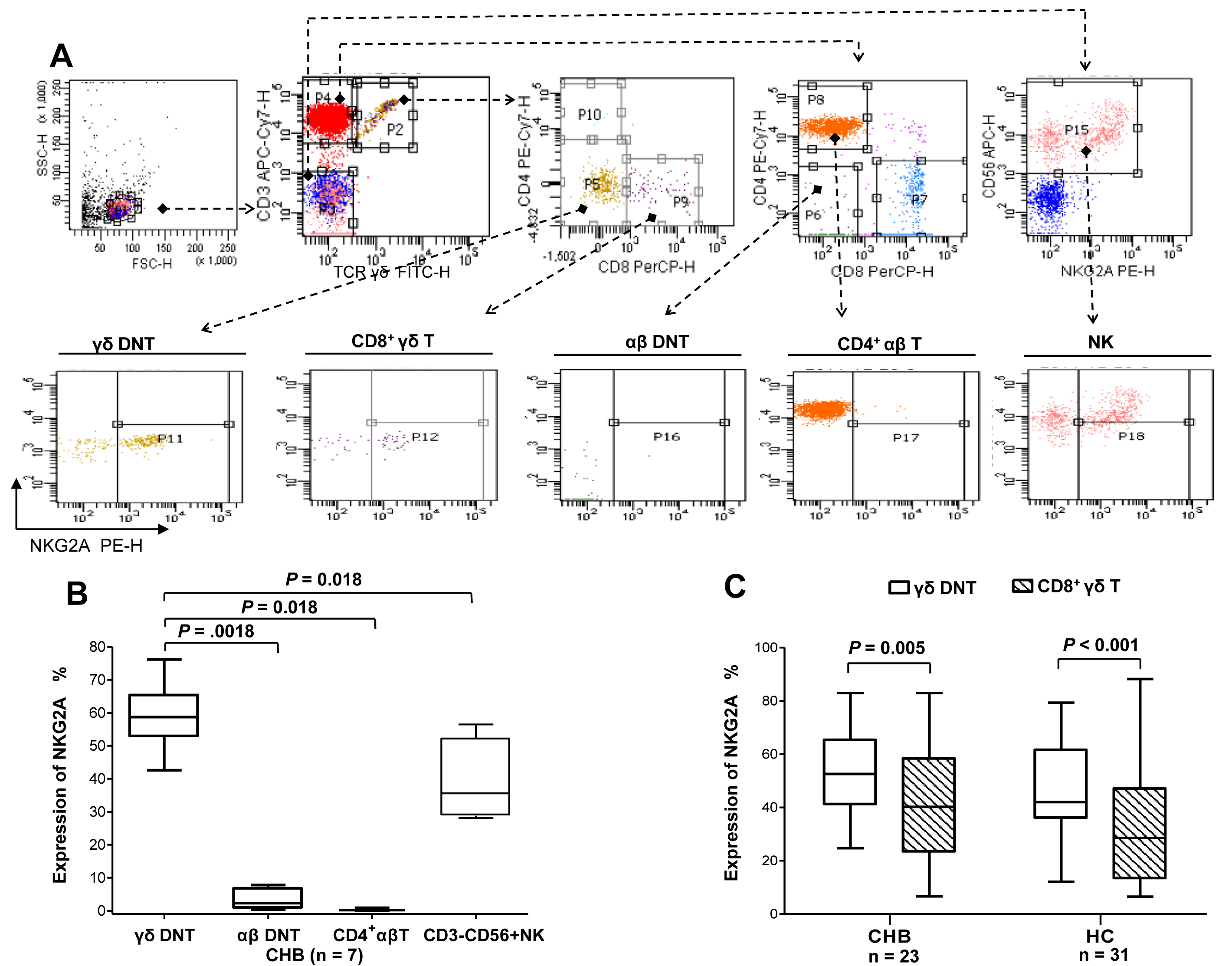

Supplement: Figure S3 — Expression of NKG2A on γδ DNT cells. PBMC from CHB patients and HC were stained with anti-CD3-APC-Cy7, anti-TCR-γδ-FITC, anti-CD4-PE-Cy7, anti-CD8-PerCP, anti-CD56-APC and anti-NKG2A-PE. (A) Expression of NKG2A on γδ DNT, CD8+γδ T, αβ DNT, CD4+αβ T cells, and NK cells were measured and compared between (B) different lymphocyte subsets or (C) between the CHB and HC groups. CHB, chronic hepatitis B; DNT cells, double-negative T cells; HC, healthy controls. (TIF) [file pone.0088475.s003.tif]

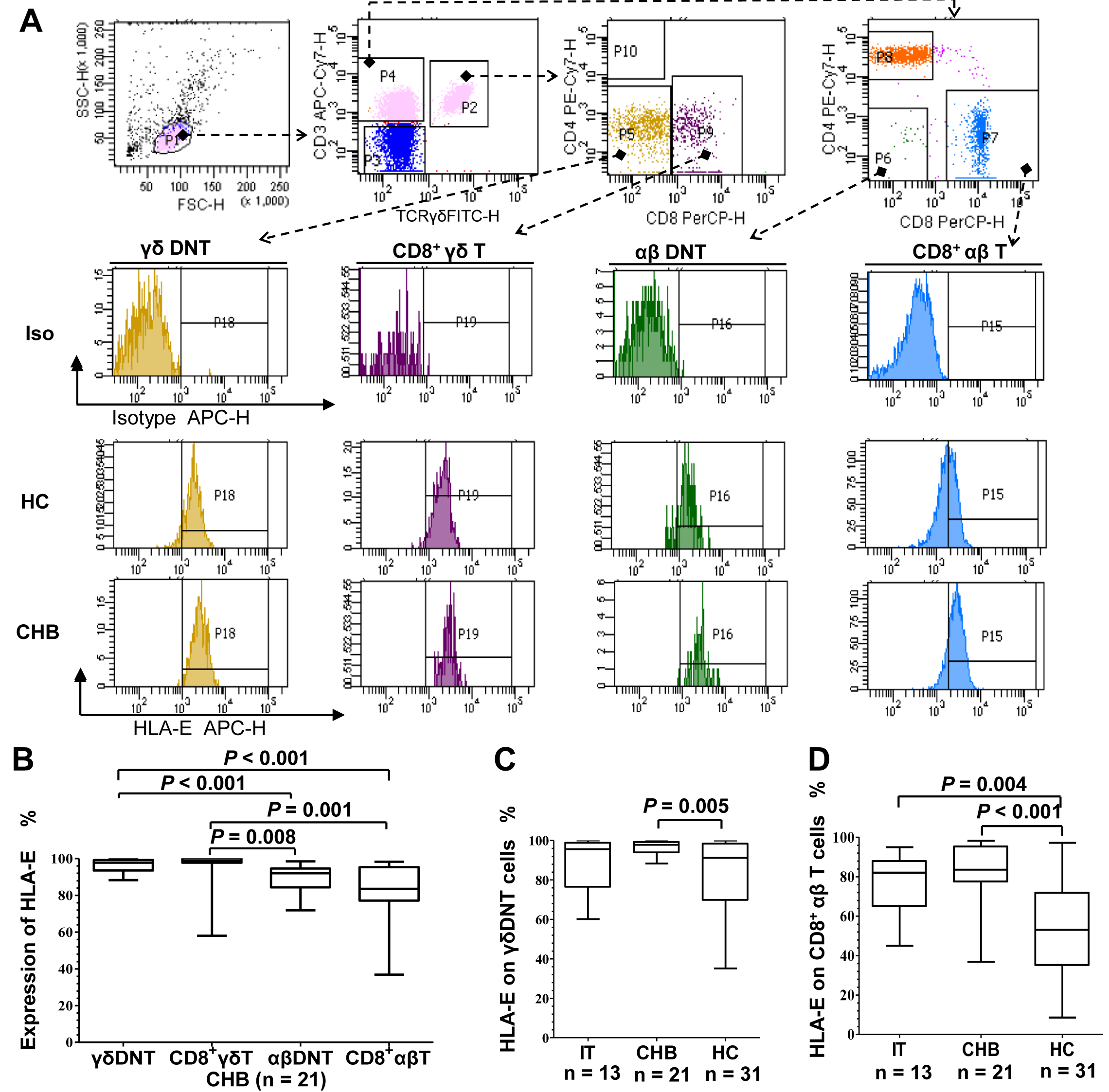

Supplement: Figure S4 — Expression of HLA-E on γδ DNT cells. PBMC were stained with anti-CD3-APC-Cy7, anti-TCRγδ-FITC, anti-CD4-PE-Cy7, anti-CD8-PerCP, and anti-HLA-APC. Expression of HLA-E on γδ DNT, CD8+ γδ T cells, αβ DNT cells, and CD8+ αβ T cells was (A) measured in HC and CHB relative to the isotype control and (B) compared in the different T-cell subsets. (C,D) Expression of HLA-E on either (C) γδ DNT or (D) CD8+ αβ T cells was compared in the IT, CHB, and HC groups. CHB, chronic hepatitis B; DNT cells, double-negative T cells; HC, healthy controls; IT, immune tolerant carriers. (TIF) [file pone.0088475.s004.tif]

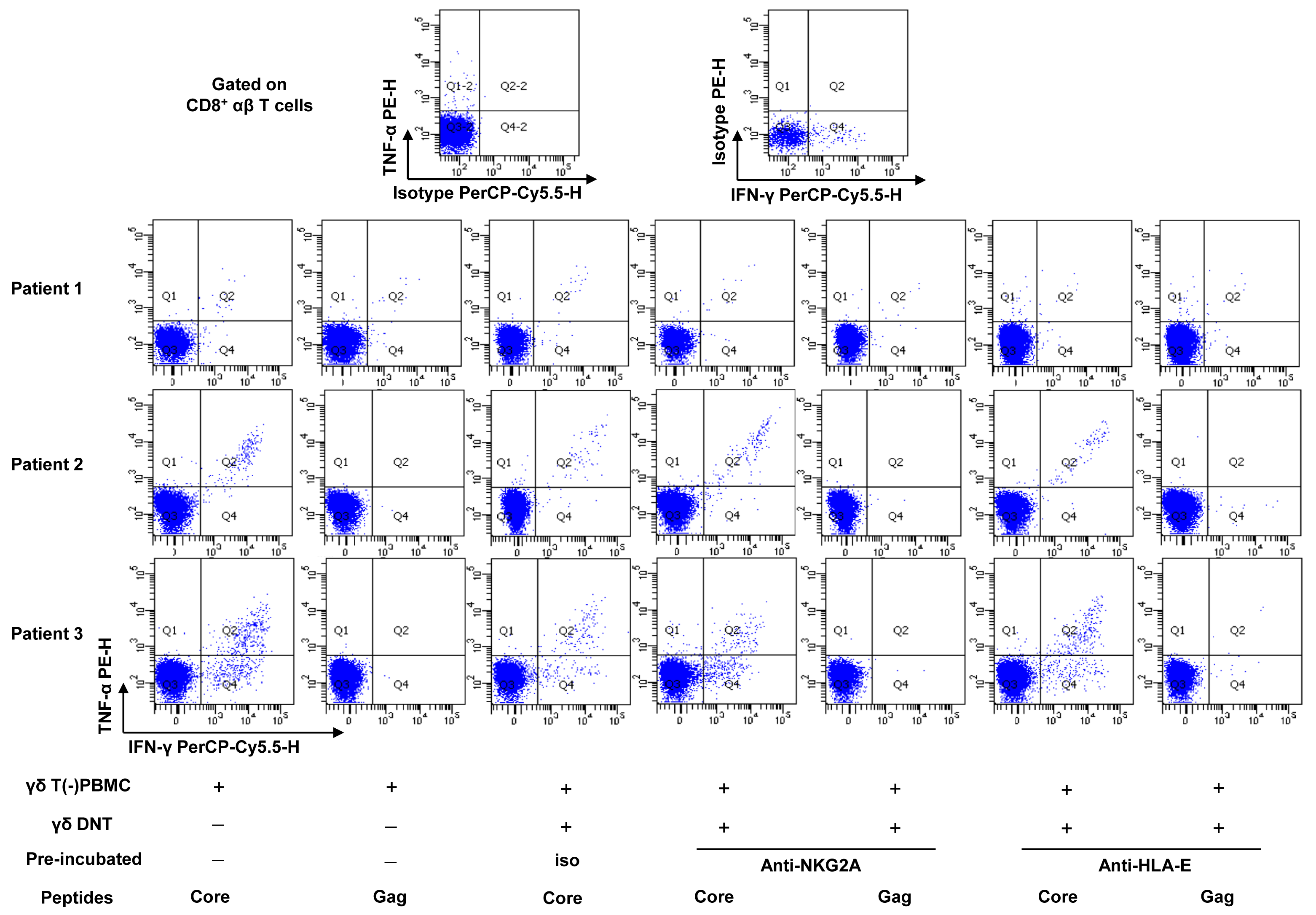

Supplement: Figure S5 — γδ DNT cell-mediated suppression of cytokine production by core peptide-stimulated CD8+αβ T cells is partially mediated by NKG2A. The plots were gated on CD8+αβ T cells. DNT cells, double-negative T cells. (TIF) [file pone.0088475.s005.tif]

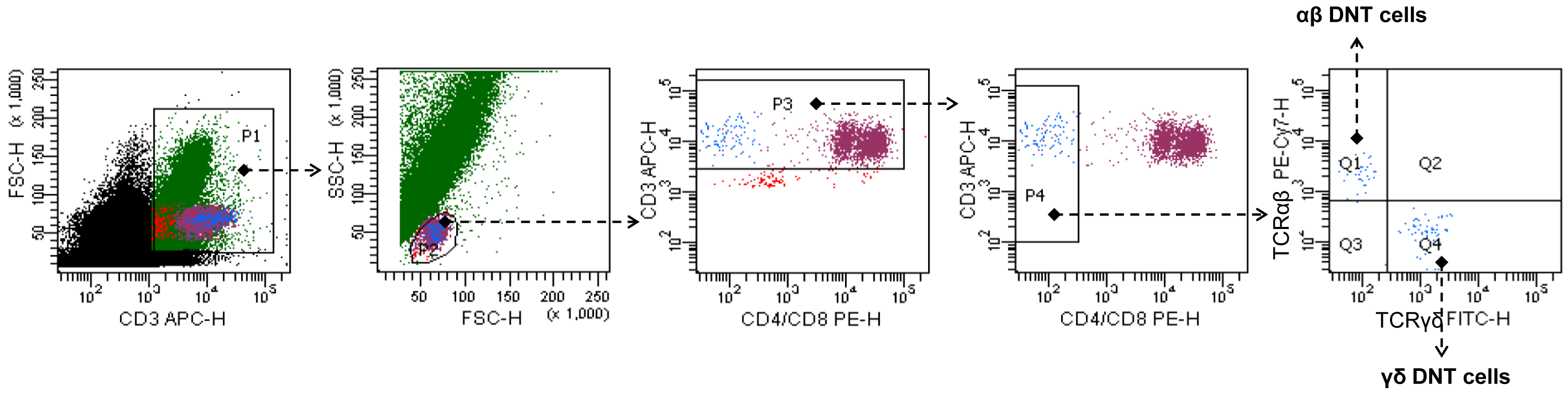

Supplement: Figure S6 — Strategy for gating the αβ DNT cells and γδ DNT cells from LIL. DNT cells, double-negative T cells; LIL, liver-infiltrating lymphocytes. (TIF) [file pone.0088475.s006.tif]
