# Supplementary material for: TCRγδ+CD4−CD8− T Cells Suppress the CD8+ T-Cell Response to Hepatitis B Virus Peptides, and Are Associated with Viral Control in Chronic Hepatitis B
Source: PLoS One. 2014 Feb 14;9(2):e88475. doi: 10.1371/journal.pone.0088475 (PMC3925121; doi:10.1371/journal.pone.0088475)
Supplement: Table S1 — The GenBank accession numbers of the sequences used to identify a panel of 26 18-mer peptides overlapping by 8 or 10 residues and covering the full HBV core open reading frame. (DOC) [file pone.0088475.s007.doc]

**Table S1.** The GenBank accession numbers of the sequences used to identify a panel of 26 18-mer peptides overlapping by 8 or 10 residues and covering the full HBV core open reading frame

| Genotype | B | C |
| --- | --- | --- |
| GenBank accession numbers | AF282918 | EF688062 |
|  | AY220703 | AY306136 |
|  | AY518556 | AY040627 |
|  | EU139543 | EU439015 |
|  | EF494382 | AY167095 |
|  | AB287328 | AB014360 |
|  | AB033554 | AY247030 |
|  | AB241116 | AF068756 |
|  | DQ448628 | EU570074 |
|  | EU57007l | DQ089795 |
