# Supplementary material for: TCRγδ+CD4−CD8− T Cells Suppress the CD8+ T-Cell Response to Hepatitis B Virus Peptides, and Are Associated with Viral Control in Chronic Hepatitis B
Source: PLoS One. 2014 Feb 14;9(2):e88475. doi: 10.1371/journal.pone.0088475 (PMC3925121; doi:10.1371/journal.pone.0088475)
Supplement: Table S2 — The amino acid sequence of core peptides. (DOC) [file pone.0088475.s008.doc]

**Table S2. The amino acid sequence of core peptides**

| Peptide number | Amino acid sequence | MW (Da) |
| --- | --- | --- |
| 1 | MQLFHLCLIISCTCPTFQ | 2098 |
| 2 | IISCTCPTFQASKLCLGW | 1971 |
| 3 | ASKLCLGWLWGMDIDPYK | 2096 |
| 4 | LWGMDIDPYKEFGATVEL | 2084 |
| 5 | EFGATVELLSFLPSDFFP | 2016 |
| 6 | LSFLPSDFFPSIRDLLDT | 2083 |
| 7 | SIRDLLDTASALYREALE | 2036 |
| 8 | ASALYREALESPEHCSPH | 1997 |
| 9 | SPEHCSPHHTALRQAILC | 2000 |
| 10 | HTALRQAILCWGELMTLA | 2027 |
| 11 | WGELMTLATWVGNNLEDP | 2046 |
| 12 | TWVGNNLEDPASRDLVVN | 1999 |
| 13 | ASRDLVVNYVNTNMGLKI | 2007 |
| 14 | YVNTNMGLKIRQLLWFHI | 2246 |
| 15 | RQLLWFHISCLTFGRETV | 2206 |
| 16 | SCLTFGRETVLEYLVSFG | 2021 |
| 17 | LEYLVSFGVWIRTPPAYR | 2167 |
| 18 | VWIRTPPAYRPPNAPILS | 2048 |
| 19 | PPNAPILSTLPETTVVRR | 1961 |
| 20 | TLPETTVVRRRDRGRSPR | 2152 |
| 21 | RDRGRSPRRRTPSPRRRR | 2318 |
| 22 | RRTPSPRRRRSQSPRRRR | 2361 |
| 23 | RRSQSPRRRRSQSRESQC | 2260 |
| 24 | FLPSDFFPSVRDLLDTAS | 2027 |
| 25 | LATWVGNNLEDPASRDLV | 1970 |
| 26 | RDLVVNYVNTNMGLKIRQ | 2133 |

MW, molecular weight.
