# Supplementary material for: TCRγδ+CD4−CD8− T Cells Suppress the CD8+ T-Cell Response to Hepatitis B Virus Peptides, and Are Associated with Viral Control in Chronic Hepatitis B
Source: PLoS One. 2014 Feb 14;9(2):e88475. doi: 10.1371/journal.pone.0088475 (PMC3925121; doi:10.1371/journal.pone.0088475)
Supplement: Table S3 — Spearman’s correlation analyses showing associations between the frequencies of γδ DNT cells and the clinical characteristics of the CHB patients at baseline n = 51). (DOC) [file pone.0088475.s009.doc]

**Table S3.** Spearman's correlation analyses showing associations between the frequencies of γδ DNT cells and the clinical characteristics of the CHB patients at baseline（n = 51）

|  | HBV DNA, log10 copies/mL | ALT, IU/L | AST, IU/L | Age, years |
| --- | --- | --- | --- | --- |
| γδ DNT cells, % | 0.263 | 0.336 | 0.913 | 0.762 |

ALT, alanine aminotransferase; AST, aspartate aminotransferase; CHB, chronic hepatitis B; DNT, double-negative T cells: HBV, hepatitis B virus.

All data are *P* values.
