# Supplementary material for: TCRγδ+CD4−CD8− T Cells Suppress the CD8+ T-Cell Response to Hepatitis B Virus Peptides, and Are Associated with Viral Control in Chronic Hepatitis B
Source: PLoS One. 2014 Feb 14;9(2):e88475. doi: 10.1371/journal.pone.0088475 (PMC3925121; doi:10.1371/journal.pone.0088475)
Supplement: Table S4 — Clinical characteristics of subjects in the cohort receiving telbivudine therapy at 104 Weeks. (DOC) [file pone.0088475.s010.doc]

**Table S4. Clinical characteristics of subjects in the cohort receiving telbivudine therapy at 104 Weeks**

| Groups | Responders | Non-responders | *P* values |
| --- | --- | --- | --- |
| Cases | 10 | 16 |  |
| Gender, M/F | 8/2 | 15/1 | 0.323 ^b)^ |
| Age, year ^a)^ | 28 (18–30) | 25 (22–27) | 0.382 ^c)^ |
| ALT, IU/L ^a)^ | 16 (13–47) | 28 (14–34) | 0.492 ^c)^ |
| AST, IU/L ^a)^ | 32 (24–36) | 28 (24–32) | 0.428 ^c)^ |
| HBV DNA,  log10 copies/mL ^a)^ | <1.85 (<1.85) | <1.85 (<1.85–3.15) | - |
| Genotype B/C | 6/4 | 7/9 | 0.344 ^b)^ |
| Knodell necroinflammatory scores ^a)^ | 5 (5) | 5 (5–6) | 0.666 ^c)^ |
| Ishak fibrosis scores ^a)^ | 2 (2–3) | 2 (1–3) | 0.402 ^c)^ |

ALT, alanine aminotransferase; AST, Aspartate aminotransferase; M/F: Male/female.

^a)^Median (IQR) M/F: Male/female

^b)^ Compared by Chi-Square Tests (Fisher’s exact test)

^c)^Compared by Mann-Whitney U test
