# Supplementary material for: TCRγδ+CD4−CD8− T Cells Suppress the CD8+ T-Cell Response to Hepatitis B Virus Peptides, and Are Associated with Viral Control in Chronic Hepatitis B
Source: PLoS One. 2014 Feb 14;9(2):e88475. doi: 10.1371/journal.pone.0088475 (PMC3925121; doi:10.1371/journal.pone.0088475)
Supplement: Methods S1 — Entry criteria for study subjects. (DOCX) [file pone.0088475.s011.docx]

**Method S1.** Entry criteria for study subjects

In the cross-sectional study, all three groups of subjects with chronic hepatitis B virus (HBV) infection were hepatitis B serum antigen (HBsAg)-positive on two occasions at least 6 months apart, and were recruited from the outpatient department of Nanfang Hospital (Guangzhou, China). All patients with chronic hepatitis B (CHB) were hepatitis B e antigen (HBeAg)-positive, had serum alanine aminotransferase (ALT) >40 IU/L on at least two occasions, and had serum HBV DNA of >100,000 copies/mL. The immune tolerant carrier (IT) group was HBeAg-positive, had normal ALT levels (ALT ≤40 IU/L) on at least two occasions during at least 1 year of follow-up, and had serum HBV DNA of >10,000,000 copies/mL. The inactive carrier (IC) group was HBeAg-negative, anti-HBe-positive, had normal ALT levels, and serum HBV DNA of <10,000 copies/mL. The healthy control (HC) group was negative for HBsAg, HBeAg, and anti-HBe, and had normal serum ALT. None of the HC subjects had a history of liver disease. All these subjects were negative for anti- HCV, anti-hepatitis E virus IgM, anti-hepatitis A virus IgM, and anti-HIV antibodies.
