# Supplementary material for: TCRγδ+CD4−CD8− T Cells Suppress the CD8+ T-Cell Response to Hepatitis B Virus Peptides, and Are Associated with Viral Control in Chronic Hepatitis B
Source: PLoS One. 2014 Feb 14;9(2):e88475. doi: 10.1371/journal.pone.0088475 (PMC3925121; doi:10.1371/journal.pone.0088475)
Supplement: Method S2 — Isolation of peripheral blood mononuclear cells and liver-infiltrating lymphocytes. (DOCX) [file pone.0088475.s012.docx]

**Method S2.** Isolation of peripheral blood mononuclear cells and liver-infiltrating lymphocytes

Peripheral blood mononuclear cells (PBMC) were isolated by Ficoll-Hypaque density-gradient centrifugation (Ficoll-Paque Plus; Amersham Biosciences, Buckinghamshire, UK), frozen in FBS containing 10% DMSO, and stored in liquid nitrogen until further use. Liver-infiltrating lymphocytes (LIL) were isolated from fragments of liver biopsy tissue that were not needed for diagnosis. Briefly, liver tissue was gently sliced into pieces 1–2 mm^3^ in size, washed twice with R10 (RPMI 1640 containing 2 mmol/L glutamine, 10% FBS, 100 U/mL penicillin, and 100µg/mL streptomycin) to remove contaminating blood, then digested in RPMI 1640 containing 500 μg/mL collagenase IV, 20 μg/mL DNase I, and 2% FBS at 37°C for 30 min. After briefly mixing by vortex, the suspension was passed through a 70-mm-nylon mesh filter (BD) to remove cell clumps and non-dissociated tissue. The filtered suspension was centrifuged (500 × *g* for 10 min at room temperature), and the cell pellet was washed twice in PBS before staining for phenotypic analysis.
